# Adenosine Receptors As Drug Targets for Treatment of Pulmonary Arterial Hypertension

**DOI:** 10.3389/fphar.2017.00858

**Published:** 2017-12-04

**Authors:** Allan K. N. Alencar, Guilherme C. Montes, Eliezer J. Barreiro, Roberto T. Sudo, Gisele Zapata-Sudo

**Affiliations:** Programa de Pesquisa em Desenvolvimento de Fármacos, Instituto de Ciências Biomédicas, Universidade Federal do Rio de Janeiro, Rio de Janeiro, Brazil

**Keywords:** pulmonary arterial hypertension, cardiopulmonary system, adenosine receptors, cell proliferation, right ventricle dysfunction, pleiotropic effects

## Abstract

Pulmonary arterial hypertension (PAH) is a clinical condition characterized by pulmonary arterial remodeling and vasoconstriction, which promote chronic vessel obstruction and elevation of pulmonary vascular resistance. Long-term right ventricular (RV) overload leads to RV dysfunction and failure, which are the main determinants of life expectancy in PAH subjects. Therapeutic options for PAH remain limited, despite the introduction of prostacyclin analogs, endothelin receptor antagonists, phosphodiesterase type 5 inhibitors, and soluble guanylyl cyclase stimulators within the last 15 years. Through addressing the pulmonary endothelial and smooth muscle cell dysfunctions associated with PAH, these interventions delay disease progression but do not offer a cure. Emerging approaches to improve treatment efficacy have focused on beneficial actions to both the pulmonary vasculature and myocardium, and several new targets have been investigated and validated in experimental PAH models. Herein, we review the effects of adenosine and adenosine receptors (A_1_, A_2A_, A_2B_, and A_3_) on the cardiovascular system, focusing on the A_2A_ receptor as a pharmacological target. This receptor induces pulmonary vascular and heart protection in experimental models, specifically models of PAH. Targeting the A_2A_ receptor could potentially serve as a novel and efficient approach for treating PAH and concomitant RV failure. A_2A_ receptor activation induces pulmonary endothelial nitric oxide synthesis, smooth muscle cell hyperpolarization, and vasodilation, with important antiproliferative activities through the inhibition of collagen deposition and vessel wall remodeling in the pulmonary arterioles. The pleiotropic potential of A_2A_ receptor activation is highlighted by its additional expression in the heart tissue, where it participates in the regulation of intracellular calcium handling and maintenance of heart chamber structure and function. In this way, the activation of A_2A_ receptor could prevent the production of a hypertrophic and dysfunctional phenotype in animal models of cardiovascular diseases.

## Introduction

Pulmonary hypertension (PH) refers to a complex group of cardiopulmonary diseases that may lead to right-sided heart failure and reduced life expectancy (Galie et al., 2016). Pulmonary arterial hypertension (PAH) represents group 1 within the PH classification system (Simonneau et al., 2013). PAH is characterized by precapillary PH (i.e., mean pulmonary arterial pressure ≥ 25 mmHg and normal pulmonary capillary wedge pressure ≤ 15 mmHg), as a consequence of exacerbated remodeling and hypertrophy of the walls of the pulmonary arteries (PAs) (Galie et al., 2016). The most severe form of PH, PAH has a prevalence of 15 cases per million adult population, incidence of 2.4 cases per million adult population per year (McGoon et al., 2013), and mean survival rate of 2.8 years in the absence of specific treatments (McGoon et al., 2013; Simonneau et al., 2013).

Current knowledge on PAH pathophysiology highlights microenvironmental changes in the pulmonary vessels as the initial cause of the disease. Crosstalk among endothelial cells (ECs), pulmonary arterial smooth muscle cells (PASMCs), myofibroblasts, pericytes, and circulating immunologic cells triggers PAH pathogenesis (Guignabert et al., 2015). Elevated pulmonary vascular resistance (PVR) causes insult to the right ventricular (RV) myocardium, with subsequent activation of neurohormonal, immunological, and mechanical-stretch signaling (Voelkel et al., 2006). The poor prognosis of PAH is mainly a consequence of long-term pressure overload in the RV chamber, which initially responds with adaptive myocardial hypertrophy. This response is followed by progressive contractile dysfunction, global heart failure, and premature death (Voelkel et al., 2006; Sztrymf et al., 2010).

Treatment of patients with PAH is palliative. Available agents include lung vasodilators, such as phosphodiesterase type 5 inhibitors, endothelin receptor antagonists, prostacyclin analogs, and guanylyl cyclase stimulators (Galie et al., 2016). Lung transplantation is an important strategy in eligible subjects with advanced PAH who are refractory to drug intervention (Galie et al., 2016). Current treatments of PAH simply control the intense PA vasoconstriction, but recent advances in understanding disease pathophysiology have driven efforts to develop new pharmacological strategies targeting the irreversible remodeling of the pulmonary vascular bed. Favorable actions in the hypertrophied and dysfunctional RV are required for better outcomes, and there is a clear need for pleiotropic targets with beneficial roles throughout the cardiopulmonary system (pulmonary vessel and cardiac cells).

Adenosine has a well-known pulmonary vasodilator effect with a rapid onset of action (Reeves et al., 1991; Nootens et al., 1995). Adenosine use is recommended as an alternative strategy in pulmonary vasoreactivity testing for identification of patients suitable for high-dose calcium channel blocker treatment (Galie et al., 2016). Patients suffering from PAH exhibit low adenosine levels in the pulmonary circulation, suggesting that this nucleoside plays a role in PAH pathophysiology. The reduction of the adenosine release on pulmonary vascular endothelium is consequent to the endothelial dysfunction that is present in PAH. Low levels in PAH may also be explained by increased activity of the adenosine deaminase in the pulmonary circulation (Saadjian et al., 1999), which was found in rats with hemolysis-associated pulmonary hypertension (Tofovic et al., 2009). However, clinical implications of the adenosine deaminase or its inhibitors in PAH remain unclear. Adenosine promotes various beneficial effects in the heart (Shryock and Belardinelli, 1997) and has been defined as a retaliatory metabolic product (Newby et al., 1985).

Long-term use of adenosine in PAH and RV failure has been limited by its extremely short half-life (Nootens et al., 1995) and adverse effects by non-selective activation of its four receptor subtypes. Nevertheless, adenosine receptors (ARs) promote multiple salutary actions in cardiac and vascular cells, including ECs, fibroblasts, and myocytes (McIntosh and Lasley, 2012; Headrick et al., 2013). Immune cells express ARs to respond to the modulatory effects of adenosine in an inflammatory environment (Hasko et al., 2008). Researchers from several fields are considering the possibility of targeting ARs as a therapeutic approach in various clinical conditions, including cerebral and cardiac diseases, sleep disorders, immune and inflammatory disorders, and cancer (Jacobson and Gao, 2006; Hasko et al., 2008; McIntosh and Lasley, 2012; Chen et al., 2013; Headrick et al., 2013).

This review compiles findings on the specific roles of each AR subtype in the cardiovascular system. The work focuses on AR activities in the small circulation, specifically in PAH pathogenesis, including the aberrant cellular proliferation and influx of inflammatory cells in and around various components of the vascular wall, as well as the distinct roles of ARs in RV cells. The main goal of this review is to answer an intriguing question: Does a specific AR exist that could be considered as a future target with pleiotropic potential in the whole cardiopulmonary system of patients with PAH?

## Pulmonary Vascular Changes in PAH

Before addressing the potential benefits of targeting specific ARs for the treatment of PAH, we must understand the intrinsic pathophysiological mechanisms of this disease. Dynamic interactions among cells in the pulmonary vascular bed are the main determinant of cellular behavior (e.g., proliferation, apoptosis, differentiation, migration, and survival) in PA walls. Abnormal cell communication can lead to development of PAH (Eddahibi et al., 2006; Xu and Mao, 2011; Guignabert and Dorfmuller, 2013; Guignabert et al., 2015). Crosstalk between PA cells is regulated through direct cell–cell contact and release of bioactive factors, such as EC-produced paracrine factors that influence the proliferation of PASMCs and fibroblasts (Eddahibi et al., 2006; Sakao et al., 2009; Amabile et al., 2013).

Below a cross-sectional diameter of 500 μm (lobular to intra-acinar level), the medial layer of the PAs is typically composed solely of smooth muscle cells (SMCs), due to loss of elastic fibers. Beyond this level, the PAs leave their adjacent airways and become barely muscularized precapillary arterioles (Guignabert and Dorfmuller, 2013). Vascular remodeling in the lungs of PAH patients initially begins in this type of arteriole and presents as PASMC hyperproliferation, underlining the importance of this cell type in PAH pathogenesis (Schermuly et al., 2011; Guignabert and Dorfmuller, 2013). Early lesions correspond to hypertrophic and hyperplastic PASMCs within the tunica media (Guignabert and Dorfmuller, 2013). The abnormal proliferation of PASMCs during PAH development occurs in response to the signaling of growth factors, including potent mitogens and chemoattractants for vascular cells, which bind to and activate surface tyrosine kinase receptors (TKRs) (Schermuly et al., 2011).

Pulmonary ECs play a crucial role during the formation and maturation of blood vessels, producing and releasing growth factors that recruit and stabilize all vascular cells (Folkman and D’Amore, 1996; Hanahan, 1997). Eddahibi et al. (2006) showed that EC-induced PASMC growth was greater in tissues from PAH patients than from controls. Dysregulation of this process and excessive release of growth factors by ECs are intrinsic abnormalities linked to PAH pathogenesis. Indeed, abnormal communication between ECs and other vascular cells in PAH may occur due to loss-of-function of the endothelium.

Among different factors, bone morphogenic protein (BMP) signaling regulates pulmonary EC survival and differentiation (Teichert-Kuliszewska et al., 2006). Patients with PAH show abnormal BMP signaling, linked to mutations of bone morphogenetic protein receptor-2 (BMPR2) in ECs (Teichert-Kuliszewska et al., 2006). These mutations could lead to deleterious consequences in ECs and PASMCs. Loss of BMPR2 signaling in ECs may increase apoptosis in response to environmental stress and injury, particularly at the level of the distal PAs (Teichert-Kuliszewska et al., 2006). This apoptosis could be an initiating mechanism for PAH by leading to vessel obliteration due to degeneration of EC structures (Zhao et al., 2005). Excessive loss of ECs promotes the development of apoptosis-resistant and hyperproliferative ECs, which are characteristic features of later stages of PAH (Voelkel et al., 2002).

The initial trigger for pulmonary EC injury is unknown, although some events, such as shear stress, local inflammation, genetic predisposition, toxins, and reactive oxygen species (ROS)-induced cell damage, might be important inducers of endothelial dysfunction (Amabile et al., 2013). Exuberant proliferation of ECs leads to formation of plexiform lesions. These dynamic networks of vascular channels formed by the monoclonal proliferation of ECs (St Croix and Steinhorn, 2016) are a morphologic hallmark of severe PAH. A single plexiform lesion can occlude the entire length of an affected vessel (Cool et al., 1999). However, debate surrounds the functional importance of these lesions in the context of PAH, as it remains unclear if they have a role in disease progression or are simply a morphologic indicator of irreversible, end-stage disease (Tuder et al., 2007; Abe et al., 2010; Jonigk et al., 2011; St Croix and Steinhorn, 2016).

In PASMCs, signaling by BMPR2 is necessary for the control of cell proliferation and differentiation (Upton et al., 2008; Garcia de Vinuesa et al., 2016). Together with EC-induced cell proliferation and medial hypertrophy, BMPR2 mutations in PASMCs participate in the process of initiating or maintaining pulmonary vessel hypertrophy in PAH patients (Zhang et al., 2003).

Fibroblasts play important roles in PAH pathogenesis by responding to injury and chemoattraction via endothelium-derived growth factors. Rapid migration of fibroblasts to the injured vessel leads to formation of the neointimal layer and, most importantly, fibroblast transdifferentiation into other cell types, including myofibroblasts – an abnormal type of PASMC that contributes to muscularization of the distal vessels (Sartore et al., 2001; Sisbarro et al., 2005; Sakao et al., 2009).

Taken together, these findings indicate that any effect on BMPR2 signaling may trigger abnormal communication between ECs, PASMCs, and fibroblasts via growth factors. Subsequently, these growth factors trigger hyperproliferation and differentiation of cells, formation of plexiform lesions, obliteration and fibrosis of the vessels, and increases of PVR and pulmonary vascular pressure.

In an elegant review, Schermuly et al. (2011) summarized the growth factors that are most clearly implicated in PAH pathogenesis. The review showed increased signaling of the following molecules in injured pulmonary vascular cells: vascular endothelial growth factor (VEGF), transforming growth factor alpha and beta (TGF-α and TGF-β), platelet-derived growth factor (PDGF), and hepatocyte growth factor (HGF) (Schermuly et al., 2011). All of these growth factors bind to TKRs, resulting in multi-phosphorylation of tyrosine residues for the assembly of downstream signaling molecules that are recruited to the receptor and activated in response to agonist stimulation (Lemmon and Schlessinger, 2010). Subsets of intracellular signaling components influenced by growth factor receptor activation are intertwined in a complex network. Examples of intracellular molecules activated by TKRs are phosphoinositide 3-kinase (PI3K)/protein kinase B (Akt), mitogen-activated protein kinases (Ras/Raf/MAPK/ERK), protein kinase C (PKC), Janus kinase (JAK), signal transducer and activator of transcription (STAT), and cyclins (a family of proteins that control cell cycle progression by activating cyclin-dependent kinases) (Koledova and Khalil, 2006; Lemmon and Schlessinger, 2010).

Roles of TKRs and their antagonists in PAH have been reviewed, and cardiac safety issues of these molecules have been raised, especially in patients with heart disease that is intrinsically related to pulmonary vascular disease (Godinas et al., 2013). The large blockage spectrum and lack of selectivity of TKR antagonists would provide unexpected toxicities, including injury to the pulmonary vasculature. Furthermore, the benefit-to-risk ratio of available TKR antagonists is low in the context of PAH (Godinas et al., 2013). Thus, new approaches for PAH treatment should be aimed at ensuring a low toxicity profile, in addition to the high anti-proliferative efficacy.

Accumulating evidence shows that RhoA, a member of the Ras homolog gene family, and its downstream effectors, the Rho kinases (ROCKs), mediate the pathogenesis of PAH through their pro-proliferative contributions (Nagaoka et al., 2006; Guilluy et al., 2009; Connolly and Aaronson, 2011). ROCK downregulates expression of anti-proliferative molecules, leading to acceleration of cell cycle progression and vascular cell hyperproliferation (Laufs et al., 1999; Sawada et al., 2000). Distal muscularization of pulmonary vessels has been linked to amplification of the RhoA/ROCK pathway in PASMCs (Emerson et al., 1999; Fagan et al., 1999; Aznar and Lacal, 2001; Keil et al., 2002).

Another important factor involved in endothelium-smooth muscle interactions, serotonin makes a major contribution to PASMC hyperplasia in PAH (Eddahibi et al., 2006). Serotonin-mediated PASMC proliferation was greater in mice with genetically mutated BMPR2 (Long et al., 2006). The BMP and serotonin mechanisms are convergent in PASMCs; both stimulate RhoA/ROCK signaling downstream for a pro-proliferative phenotype (Liu et al., 2009). These previous reports suggest that vascular cell proliferation induced by downstream growth factors could be attenuated, in part, through inhibition of RhoA/ROCK signaling.

The RhoA/ROCK pathway is of interest to pharmaceutical companies due to the pleiotropic effects of its inhibitors in numerous diseases (Feng et al., 2016). Fasudil is a ROCK inhibitor that has shown salutary effects in the cardiopulmonary system of PAH patients and in different experimental models of the disease. These effects derive from its anti-proliferative profile in the pulmonary vessels, which it achieves by reducing PVR and RV overload (Mouchaers et al., 2010; Raja, 2012; Fukumoto et al., 2013; Jiang et al., 2014; Xiao et al., 2015; Gupta et al., 2017). Nevertheless, the cardioprotective potential of this pharmacological modality is not completely clear, as the effect of fasudil on reducing PAH-related changes in the RV may be an indirect response of the lower afterload in the right heart chamber. In addition to the beneficial effects of ROCK inhibitors in the left heart (Demiryurek et al., 2005; Takeshima et al., 2012), their direct effects should be investigated in the RV cells, because the structures, hemodynamics, and functions of both ventricles are distinct.

Secondarily to the pro-proliferative phenotype in pulmonary vascular cells, damaged crosstalk between ECs and PASMCs can exacerbate PA vasoconstriction as a consequence of an imbalance in the production of endothelium-derived vasodilator [nitric oxide (NO) and prostacyclin] and constrictor factors (endothelin-1, serotonin, and angiotensin II). Crosstalk dysfunction is a pivotal element in the development and progression of the disease (Guignabert et al., 2015).

Furthermore, PAH is among many pathophysiologic conditions that are linked to inflammation (Schermuly et al., 2011). Inflammation has a pivotal role during development of PAH in humans and in animal models of the disease. Some patients with PAH present increased levels of tumor necrosis factor alpha (TNF-α), interleukins 12 and 6 (IL-12 and IL-6), and interferon-γ, associated with other inflammatory signals, such as plasma cell dyscrasia polyneuropathy. Induction of PAH in rodents by chronic hypoxia or monocrotaline leads to an increase in the number of inflammatory cells in the lungs (Minamino et al., 2001; Soon et al., 2010; Bi et al., 2013). Vascular and inflammatory cells are important local sources of cytokines and chemokines, which can trigger pulmonary vascular remodeling in PAH (Sutendra et al., 2011; Rabinovitch et al., 2014). Histopathological studies have demonstrated the presence of complement system components, autoantibodies, and inflammatory cells (neutrophils) in the vessel lumen, which can bind to the endothelium and may infiltrate the medial muscular layer. Inflammatory infiltrate in the neointimal layer is composed of T- and B-lymphocytes, with macrophages, mast cells, and dendritic cells present in the adventitial layer. Lymphoid follicles, characterized by T cells, B cells, and plasmacytoid dendritic cells (APCs), are found in the periadventitial space (Stacher et al., 2012; Rabinovitch et al., 2014). Thus, reversion of the pulmonary vessel injury in PAH requires an anti-inflammatory profile.

## Cardiovascular Functions of Adenosine and its Receptors

Adenosine is a purine nucleoside that is widely distributed in all tissues. Structurally, adenosine is formed by the linking of adenine to a ribose sugar molecule. Synthesis of adenosine is increased after myocardial infarction (MI) (Daly, 1982; Hasko and Cronstein, 2004; Asakura et al., 2007; Hisatome, 2007). Adenosine is synthesized inside and outside the cells for immediate use and is not stored in vesicles for future release. For these reasons, this molecule is an autacoid, similar to several biological transmitters (Newby and Holmquist, 1981; Deussen et al., 1989; Deussen and Schrader, 1991; Decking et al., 1997; Zimmermann, 2000).

Extracellular production of adenosine depends on a cascade reaction initiated by ATP and ADP as substrates (Dunwiddie et al., 1997). ATP and ADP are converted to AMP by catalysis of ectonucleoside triphosphate diphosphohydrolases (NTPDases 1, 2, 3, and 8) on the cell surface, followed by AMP hydrolysis to adenosine by ecto-5′-nucleotidase (NTE5/CD73) (Ballard, 1970; Headrick and Willis, 1990; Decking et al., 1997; Bak and Ingwall, 1998; Gustafson and Kroll, 1998). Intracellular adenosine might be provided through other signaling processes. One of these events is the catabolism of ATP from stressed cells under hypoxia (Ham and Evans, 2012). This condition might be responsible for the perturbed recycling of adenosine and ATP, and the increased activity of specific phosphatases leads to excessive levels of adenosine (Spychala, 2000). Another source of intracellular adenosine is the cytosolic 5′-nucleotidase-mediated metabolism (dephosphorylation) of AMP, which is formed from the degradation of cyclic AMP (cAMP) by phosphodiesterases. Intracellular adenosine is also importantly generated by the conversion of *S*-adenosylhomocysteine to adenosine and homocysteine, catalyzed by *S*-adenosylhomocysteine hydrolase (Kloor and Osswald, 2004; Hermes et al., 2007; Antonioli et al., 2015).

Adenosine is converted, extra- or intracellularly, to inosine by adenosine deaminase (Ford et al., 2000; Singh and Sharma, 2000; Antonioli et al., 2012). Phosphorylation of adenosine to AMP by adenosine kinase reduces the intracellular concentration of adenosine (De Jong, 1977; Drabikowska et al., 1985; Pak et al., 1994; Boison, 2013). The adenosine concentration depends on two families of transmembrane proteins, concentrative and equilibrative nucleoside transporters, which facilitate the movement of nucleosides, nucleobases, and their analogs across the cell membrane. There are four isoforms of human equilibrative nucleoside transporters, but only ENT1 and ENT2 are expressed in tissues throughout the body (King et al., 2006; Playa et al., 2014). The adenosine transport process usually follows gradients from high to low concentration, with no energy spending (Chen et al., 2013). The extracellular adenosine concentration varies substantially between tissue types (Ramakers et al., 2008). The local adenosine concentration may be increased from the nano- to the micromolar range, signaling tissue injury, by extreme pathophysiological cell activation (seizure) or insults due to ischemia, trauma, inflammation, or cancer (Fredholm et al., 2001; Linden, 2001; Flamand et al., 2006; Hasko et al., 2008; Antonioli et al., 2012, 2013; Hasko and Cronstein, 2013).

Drury and Szent-Gyorgyi (1929) described adenosine as a potent vasodilator-like molecule. In the cardiovascular system, adenosine offers protective effects against episodes of angina pectoris, preconditioning, and ischemia/reperfusion (I/R) injury (Olafsson et al., 1987; Lasley and Mentzer, 1992; Woolfson et al., 1996; Miura et al., 2000). Adenosine mediates its physiological effects on tissue regeneration and repair by binding to and activating a family of G-protein coupled receptors, denoted P1 purinoceptors and adenosine receptors A_1_, A_2A_, A_2B_, and A_3_ (Ralevic and Burnstock, 1998; Merighi et al., 2015), all expressed in the cardiovascular system (Headrick et al., 2013).

The transduction pathways involved in adenosine signaling through its receptors in the cardiovascular system are complex. A_1_ receptor may couple to different G-proteins in order to regulate adenylyl cyclase (AC), phospholipase C (PLC), Ca^2+^ and K^+^ channel functions, and possesses a high affinity for adenosine (Fredholm et al., 2000). When it is coupled to Gi protein it inhibits AC and modulates K^+^ and Ca^2+^ channels activities. But it may also couple with Gs to activate AC, or with Gq/11 to stimulate PLC enzyme and, subsequently, the inositol triphosphate (IP_3_) and diacylglycerol (DAG) production (Headrick et al., 2013). It was also described that A_1_ receptor promotes K^+^ efflux via βγ subunit of G-protein inwardly rectifying channels (GIRK/K_IR_3) (Kurachi et al., 1986; Kirsch et al., 1988; Belardinelli et al., 1995; Mubagwa and Flameng, 2001).

The A_2A_ receptor is very sensitive to adenosine (Fredholm et al., 2000). This receptor couples with Gs to activate AC and increase cAMP levels in the cardiovascular system and it interacts with A_1_ receptor, dopamine D_2_, metabotropic glutamate 5, NMDA and cannabinoid CB_1_ receptor in other organs, but the specific functions and occurrence of such interactions remains unknown (Headrick et al., 2013).

Adenosine has the lowest affinity for the A_2B_ receptor subtype (Fredholm et al., 2000). A_2B_ signaling is mediated via Gs protein to stimulate AC activity, but it also may couple to Gq/11 protein to activate PLC (Headrick et al., 2013). It was reported that A_2B_ receptor might also modulate the arachidonic acid cascade (Donoso et al., 2005). Additionally, A_2B_ signaling can increase endothelial NO bioavailability with subsequent K_ATP_ channel opening in rodent vascular cells (Morrison et al., 2002; Hinschen et al., 2003). A_2B_ may also couple to p38-MAPK in coronary vessels (Teng et al., 2005).

The A_3_ receptor is the newest subtype that was identified (Fredholm et al., 2000). Similarly to A_1_ receptor, it can couple to Gi protein to inhibit AC activity, and also to Gq/11 to modulate PLC and Ca^2+^ intracellular handling (Headrick et al., 2013). Involvement of A_2A_ and A_2B_ receptor-mediated vasodilation has been reported in several vessels, including the muscular arteries [mesenteric (Hiley et al., 1995), renal (Rump et al., 1999), and coronary arteries (Flood and Headrick, 2001)] and elastic arteries, including the aorta of several species [guinea pigs (Stoggall and Shaw, 1990), rats (Prentice and Hourani, 1996), and hamsters (Prentice and Hourani, 2000)]. Adenosine relaxes precontracted isolated PA rings, an effect that probably occur via A_2A_ and A_2B_ receptor activation (El-Kashef et al., 1999). Some investigators have suggested that vascular relaxation in response to A_2A_ activation may be independent of ECs (Pearl, 1994), whereas others have shown substantial EC involvement (Martin and Potts, 1994). Resolving this controversy, researchers demonstrated that A_2A_ is located in both the vascular endothelium and vascular SMCs (Leal et al., 2008), and that its activation is involved in vasodilation (Lewis et al., 1994; Prentice and Hourani, 1996).

Activation of the Gs protein-coupled endothelial A_2A_ receptor triggers NO release by activating the AC-protein kinase A (PKA) pathway (Ikeda et al., 1997; Ray and Marshall, 2006). Activation of A_2A_ in vascular SMCs increases formation of cAMP and activation of PKA, which leads to phosphorylation and opening of potassium channels. This effect, in turn, causes hyperpolarization and vasodilatation (Ko et al., 2008). In contrast, the A_1_ and A_3_ receptors negatively modulate A_2A-B_ receptor-induced vasodilation (Talukder et al., 2002; Mustafa et al., 2009; Ponnoth et al., 2009). The A_1_ receptor plays a negative role in regulating blood pressure, causes contraction of vascular smooth muscle, and decreases coronary blood flow (Ponnoth et al., 2009). These findings indicate that adenosine can act as a vasoconstrictor or a vasodilator, depending on its interaction with specific receptor subtypes, plasma levels, and tissue localization. Differences in the adenosine regulation of the pulmonary vascular tonus might be explained, in part, due to the higher affinity of this nucleoside for the A_1_ receptor (promotes pulmonary vasoconstriction) than for A_2B_ receptor (promotes pulmonary vasodilation). Saadjian et al. (1999) have described a correlation of the progression of PH and the lower level of adenosine observed in patients. Low adenosine levels in lungs from PH patients are likely to stimulate A_1_ more than A_2B_ receptors, thus contributing to the vasoconstriction in those subjects.

The pulmonary circulation is one of the few regions in the body where activation of ARs has a dual function (i.e., promoting both contraction and relaxation) that depends on the basal tone of the blood vessel (Cheng et al., 1996). In pulmonary vessels, A_1_ receptors are stimulated under circumstances of low vasculature tone, thereby promoting vasoconstriction; when vessel tone is high, A_2_ receptor subtypes are activated and promote vasodilation (Tabrizchi and Bedi, 2001). We can extrapolate these findings to the context of PAH. In terms of the induction of pulmonary vessel relaxation, ARs in the large PAs (vs. small arteries) were reported to be the A_2B_ subtype (vs. A_2A_ subtype) (Tabrizchi and Bedi, 2001). This information is relevant in the pathological environment of PAH, as the disease begins in the distal microvasculature. Thus, we may assume that activation of A_2_ receptor subtypes in the pulmonary vessels could be targeted to reduce the extremely high vascular tone in PAH patients.

Pharmacological manipulation of adenosine signaling is of great interest for numerous cardiovascular conditions. Several potential agonists or antagonists of ARs are being studied in experimental models of left ventricle (LV) ischemia and dysfunction and systemic hypertension. Whether the development of selective and potent ligands for ARs could be an interesting treatment alternative in the context of PAH and RV failure remains speculative.

## Roles of ARs in Proliferation of Pulmonary Vessel Cells

Repair of the pulmonary vessel cells in patients with PAH could be promoted by inhibition of the migration and/or proliferation of ECs, PASMCs, and/or fibroblasts. In this section, we describe recent findings for each AR subtype regarding their roles as targets to control PA wall remodeling and subsequent hypertrophy.

Several studies demonstrated the presence of ARs in lung in different species. For example, A_1_, A_2B_, and A_3_ receptors were detected in rat airway SMCs (Michoud et al., 2002). In humans, levels of A_2B_ transcripts were the highest in bronchial SMCs; A_1_ and A_2A_ transcripts were detected as well, but A_3_ transcripts were below the detection limit (Zhong et al., 2004). Immunohistochemical analyses of human lung parenchyma demonstrated expression of A_2A_ and A_3_ receptors in bronchiolar and alveolar epithelial cells, SMCs in bronchiolar and vessel walls, and ECs in the PAs (Varani et al., 2006). In contrast, A_2B_ receptor was expressed only in mast cells and macrophages, and A_1_ receptor was expressed only in a few alveolar macrophages (Varani et al., 2006). Differences in AR expression between rodents and humans might be due to the cell- and tissue-specific effects of hypoxia on their expression (Fozard and Hannon, 2000).

Few studies have addressed specific roles of the A_1_ receptor in the growth regulation of pulmonary vascular cells. This receptor subtype is down regulated in the pulmonary *vasa vasorum* ECs of calves with experimentally induced neonatal PH and in these cells the A_1_ receptor activation leads to actin cytoskeletal remodeling and a barrier formation in *vasa vasorum.* A_1_ activation in ECs could be targeted with the goal of reducing neovascularization and function of the *vasa vasorum*, indirectly contributing to the integrity of the pulmonary vasculature by preventing the triggering of inflammation (Umapathy et al., 2013). Nevertheless, A_1_ receptor could be only a vascular bed-specific target for PAH in advanced stages, by blocking *vasa vasorum* expansion in large pulmonary vessels. Although the knowledge of the influence of A_1_ receptor in animal model of PAH, this receptor is poorly expressed in human pulmonary vascular cells (Varani et al., 2006). Thus, it could be considered that A_1_ receptor may not be relevant to the progression of HAP, but it is important further evaluation to characterize specific functions of this adenosine receptor subtype in the small lung vasculature from PAH patients, since in some pathological conditions the adenosine receptors pattern might be changed.

A_2A_ is the most well-described AR subtype in the pulmonary circulation and in the context of PAH. Using an A_2A_ receptor knockout (KO) mouse model, Xu et al. (2011) provided the first evidence of the critical contribution of A_2A_ to PAH development. At a postnatal age of 14–16 weeks, A_2A_ KO mice exhibited hemodynamic, histological, and ultrastructural characteristics suggestive of PAH. These changes included increases in RV systolic pressure, RV mass, and wall area and thickness, cellular proliferation in pulmonary resistance vessels, activation and hypertrophy of the PASMCs and ECs, and collagen deposition in the PA wall adventitia (Xu et al., 2011). The spontaneous PAH and altered PA remodeling were supported by the anatomical localization of A_2A_ in the vasculature, further demonstrating the functional activation of A_2A_ in ECs. These findings suggest that the effect of adenosine in PAH is likely mediated by the A_2A_ receptor in pulmonary vessels (Xu et al., 2011).

Recently, the same research group showed that A_2A_ KO mice exhibited key pathogenic characteristics of PAH, including muscularization of the pulmonary arterioles, PA remodeling, lumen narrowing, proliferation of pulmonary vascular ECs and SMCs, excessive hypertrophy of fibroblasts, and collagen deposition. A_2A_ KO mice overexpressed RhoA and ROCK mRNA and protein. As mentioned above, activation of RhoA/ROCK signaling may cause pulmonary vascular remodeling and development of PAH. Thus, this experimental study provides sufficient evidence for validation of the A_2A_ receptor as an anti-remodeling target in the pulmonary circulation (Shang et al., 2015). As such, this receptor may be a promising target for PAH therapy in the future (Antoniu, 2012). We agree with the authors on the need to confirm the specific downstream biochemical pathways that lead to inhibition of RhoA/ROCK signaling by A_2A_ receptor activation.

Salidroside, an active ingredient isolated from *Rhodiola rosea*, has multiple pharmacological activities, including anti-inflammatory, antioxidation, antistress, anticancer, and immune-enhancing effects. Salidroside effectively inhibited chronic hypoxia-induced PAH and PA remodeling by increasing A_2A_ expression and enhancing A_2A_-related mitochondria-dependent apoptosis. The authors suggested that it could be targeted for reducing vessel wall remodeling and hypertrophy in PAH (Huang et al., 2015).

New compounds of the class *N*-acylhydrazones were designed and synthetized to act as agonists of AR. The lead compound for these new derivatives was named LASSBio-294, which showed beneficial activity in rat model of MI (Costa et al., 2010; da Silva et al., 2014) via activation of the A_2A_ receptor (da Silva et al., 2017). LASSBio-294 has shown low efficacy in the rat model of PAH (data not published) which leads to explore the influence of adding the methyl group in the *N*-acylhydrazone function as a chemical strategy to optimize its pharmacological properties. Thus, among the new series of methylated *N*-acylhydrazone derivatives, LASSBio-1359 (EC_50_ = 6.6 ± 1.7 × 10^-6^ M) (Alencar et al., 2013) and LASSBio-1386 (EC_50_ = 6.8 ± 0.6 × 10^-6^ M) (Alencar et al., 2014), showed a more potent vasodilator effect in rat PAs than LASSBio-294 (EC_50_ = 7.0 ± 0.7 × 10^-2^ M). Those data reinforced the fact that the *N*-methyl group markedly changes the conformation of *N*-acylhydrazone function in the pulmonary vascular target. It was also previously described the importance of the presence of a small lipophilic group in substitution of the amide group of the *N*-acylhydrazone moiety to the increased cardiovascular effects (Silva et al., 2005).

Both compounds, LASSBio-1359 and LASSBio-1386, promoted PA vasodilation by the activation of the A_2A_ receptor, since their vascular effects were reduced by the selective antagonist of the A_2A_ receptor, ZM 241385 (Alencar et al., 2013, 2014). The binding assays for both substances confirmed their higher selectivity for the A_2A_ receptor subtype than for others (e.g., A_1_, A_2B_, and A_3_). LASSBio-1359 (10 μM), significantly inhibited the binding of agonist to the A_2A_ receptor (CGS21680) by an average of 78.6%, while for other receptors it did not surpass 30% inhibition (Alencar et al., 2013). Furthermore, computational docking studies demonstrated how LASSBio-1359 and LASSBio-1386 might interact with the amino acid residues of the A_2A_ receptor crystal structure compared to the agonist CGS21680, supporting the fact that they were ligands of A_2A_ receptors (Alencar et al., 2013, 2014).

The involvement of the A_2A_ receptor in the regulation of cardiopulmonary physiology was investigated after administration of LASSBio-1359 and LASSBio-1386 in rats with monocrotaline-induced PAH (Alencar et al., 2013, 2014). Animals with monocrotaline-induced PAH, exhibited intense pulmonary microvessel remodeling and hypertrophy and when treated with LASSBio-1386 showed reductions of the proliferative changes in the pulmonary arterioles and pulmonary vascular remodeling. It was also observed a downregulation of the A_2A_ receptor in pulmonary tissue and RV tissue from rats with PAH (Alencar et al., 2014). Another A_2A_ receptor ligand, LASSBio-1359, induced pulmonary vascular relaxation and promoted recovery of endothelial function in PA rings from rats with PAH. Monocrotaline-induced PAH produced fibromuscular hypertrophy and hyperplasia in the arteriole walls which in turn increased the RV systolic pressure and led to RV hypertrophy. However, daily oral treatment with the A_2A_ agonist abolished the increased RV overload and reduced vessel wall hypertrophy. Importantly, LASSBio-1359 exhibited satisfactory efficacy through long-term oral administration regimens in the cardiopulmonary system of rats with PAH, with no side effects in the systemic circulation, such as hypotension or a compensatory increase in heart rate (Alencar et al., 2013).

**FIGURE 1:**
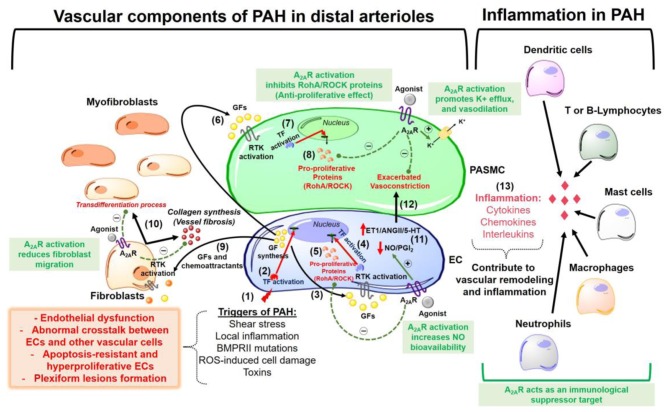
Overview of pathophysiological mechanisms responsible for pulmonary arterial hypertension (PAH) development in lung pre-capillary arterioles. The beneficial effects of the adenosine A_2A_ receptor activation are highlighted in the green boxes. The events are sequentially numbered: (1) Triggers of PAH initially promote endothelial cells (EC) injury and dysfunction. The apoptosis-resistant ECs develop a hyperproliferative phenotype with a subsequent formation of obstructive plexiform lesions and this might be explained by (2) activation of transcription factors (TF) that promote a nuclear codification for an abnormal synthesis of growth factors (GF) and chemoattractants. (3) These GFs act activate receptors tyrosine kinase (RTK) in the ECs (autocrine signaling). (4) activation of RTK in ECs promotes gene transcription for the synthesis of (5) pro-proliferative proteins (e.g., RhoA/ROCK proteins). (6) ECs also release GFs that act in the pulmonary arterial smooth muscle cells (PASMC) triggering a paracrine signaling which is similar to what happens after activation of RTKs in ECs (steps 7 and 8) for the synthesis of pro-proliferative proteins. (9) Endothelial-derived chemoattractants and GFs also are responsible by fibroblasts migration and (10) collagen deposition (vessel wall fibrosis) with subsequent transdifferentiation into myofibroblasts. Other pathogenic characteristics of PAH are (11) increased endothelial production of vasoconstrictor molecules [endothelin-1 (ET-1), angiotensin II (AngII) and serotonin (5-HT)] and decreased release of vasodilators as nitric oxide (NO) and prostacyclin (PGI_2_), an event that is responsible by the (12) exacerbated vasoconstriction in the disease progression. (13) An inflammatory environment is formed by recruitment of several immunological cells, all of which releasing molecules as cytokines, chemokines, and interleukins that contribute to vascular remodeling and lung vessel injury through inflammation. A_2A_R, adenosine A_2A_ receptor; ROS, reactive oxygen species; RhoA/ROCK, pro-proliferative Rho kinases.

Stimulation of the A_2A_ receptor effectively reduced neointimal layer formation in a murine model of carotid artery ligation (McPherson et al., 2001). Collagen deposition and wall thickening were increased in the adventitial layer of PA walls from A_2A_ KO mice (Xu et al., 2011). Therefore, we speculate that in addition to its anti-inflammatory effects (addressed later in this review), A_2A_ exhibits anti-remodeling activity in the pulmonary vascular bed, through which it can reduce fibroblast migration and transdifferentiation into myofibroblasts during PAH pathogenesis. This possibility was supported by our study showing that the A_2A_ receptor agonist LASSBio-1359 reduced collagen deposition in the pulmonary arterioles of PAH rats (Alencar et al., 2013). **Figure 1** shows an overview of the beneficial effects of A_2A_ receptor in the pathogenesis of PAH.

Of the four ARs, A_2B_ has emerged as the receptor that regulates many of the adenosine-driven remodeling responses seen in chronic lung diseases (Karmouty-Quintana et al., 2013b). Although there have been no studies of the A_2B_ receptor in *in vivo* animal models or clinical PAH, there have been some studies of its roles in PH due to lung fibrosis or chronic obstructive pulmonary disease (COPD).

Pulmonary hypertension is a common and deadly complication of interstitial lung disease (Behr and Ryu, 2008). Genetic removal of the A_2B_ receptor or treatment with its selective antagonist attenuated vascular remodeling in a mouse model of PH related to lung fibrosis. Karmouty-Quintana et al. (2012) proposed that A_2B_ receptor activation can promote the release of endothelin-1 and IL-6 from ECs and PASMCs, respectively, potentiating vessel wall remodeling and evolution to a PH phenotype. These authors later demonstrated an upregulation of the adenosine axis in lungs from patients with PH secondary to idiopathic pulmonary fibrosis, leading to enhanced accumulation of adenosine and expression of A_2B_. The authors stated that, under acute conditions, hyperactivation of the A_2B_ receptor by adenosine is protective and leads to lung tissue repair. In humans with lung fibrosis, however, sustained activation of the receptor is deleterious and contributes to development of pulmonary vascular remodeling and PH (Garcia-Morales et al., 2016).

Varani et al. (2006) reported that the A_2B_ receptor is expressed only in mast and macrophage immunologic cells of human lung. Recently, researchers found that conditional deletion of the A_2B_ receptor from myeloid cells was able to alter the phenotype of macrophages and dampen the development of fibrosis in a mouse model of lung injury-induced PH. The authors suggested a role for A_2B_-expressing myeloid cells as important regulators of fibrosis. These findings open the possibility of selectively blocking this AR subtype on macrophages as a novel therapeutic strategy for lung fibrosis and PH secondary to chronic lung diseases (Karmouty-Quintana et al., 2015).

Patients with COPD frequently develop PAH, characterized by extensive remodeling of the pulmonary vasculature due to increased proliferation of PASMCs and ECs, muscularization of previously non-muscular arteries, increased vascular tone, and formation of complex vascular lesions. Alveolar hypoxia, inflammation, and emphysema in patients with COPD contribute to remodeling, vasoconstriction, and reduction of the vascular bed (Chaouat et al., 2008). Patients with both COPD and PH present remodeled vessels, characterized by increased smooth muscle and collagen deposition. Patient lung sections had elevated A_2B_ transcript levels, which were significantly correlated with increased PA pressures. Findings from this study suggest a role for A_2B_ receptor antagonism as treatment for PH secondary to COPD (Karmouty-Quintana et al., 2013a).

On the other hand, in an *in vitro* model of cultured PASMCs under hypoxic conditions, Qian et al. (2013) showed that activation of the A_2B_ receptor induced antiproliferative effects. In another report, A_2B_ receptor stimulation counteracted PDGF-induced proliferation of human coronary SMCs through activating the exchange protein activated by cAMP (Mayer et al., 2011). Antiproliferative effects mediated by A_2B_ receptors have been observed in studies of rat and human aortic and pre-glomerular SMCs and in human coronary SMCs (Dubey et al., 1996, 1998, 2010; Jackson et al., 2010, 2011). In aortic-cultured SMCs, the proliferation-inducing transcription factor B-Myb elevated endogenous A_2B_ receptor mRNA and receptor activity levels, which, in turn, decreased cell proliferation (St Hilaire et al., 2008). Accordingly, we assume that it would be of great importance to evaluate the specific roles of the A_2B_ receptor subtype and its agonists in both clinical and pre-clinical PAH experiments for further considerations about using this receptor as a target for the disease treatment. What would be an innovative strategy, antagonism or agonism of the A_2B_ receptor in PAH patients? This question might give a clue for the basic and clinical science about discovering any beneficial strategy concerning A_2B_ receptor for the treatment of PAH and its symptoms.

Regarding the specific roles of the A_3_ receptor in the proliferation of pulmonary vessel cells in the context of PAH, we did not find consistent studies to discuss in this review. Nevertheless, experiments performed in aortas from A_3_ receptor-deficient mice showed that this receptor has a role in increasing the proliferation of vascular SMCs (Jones et al., 2004). Another study demonstrated that A_3_ receptor activation in cultured human coronary SMCs was coupled to SMC proliferation via activation of phospholipase C and MAPK, with subsequent induction of the early growth response proteins EGR2 and EGR3 (Hinze et al., 2012).

## Roles of ARs in Inflammation

There is divergent information regarding the A_1_ receptor in the inflammatory process. In several animal models of inflammation, A_1_ receptor mediated anti-inflammatory effect (Liao et al., 2003; Lee et al., 2004; Tsutsui et al., 2004; Kim et al., 2008), however, A_1_ receptor is also implicated with altered vascular response and systemic inflammation in an allergic mouse model of asthma (Ponnoth et al., 2010). Moreover, the block of A_1_ receptor attenuated endotoxin-induced lung injury in cats (Neely et al., 1997). Immune cells appear to exert activity mediated by activation of the A_1_ receptor because its activation may promote neutrophils adherence to endothelial cell and chemeotaxis (Barletta et al., 2012). Neutrophils are cells recruited to tissue in response to pathogen (host defense) or some inflammatory disease (Barletta et al., 2012). In early stage of inflammation the concentration of adenosine is low which may evoke neutrophil recruitment (Cronstein et al., 1990).

Evidences have suggested that anti-inflammatory effect via activation of A_2A_ receptor is a result of a positive stimulation of adenylate cyclase (AC) system with increased PKA levels and subsequently decrease NF-κB signaling through lower release of pro-inflammatory cytokines, such as TNF-α and IL-1β. Moreover, to decrease the release of interleukins and chemokines, the A_2A_ receptors activation inhibits events that occur during an immune response such as antigen presentation, adhesion and trafficking cellular, cell proliferation (Milne and Palmer, 2011; Headrick et al., 2013; Geldenhuys et al., 2017). A_2A_ receptors are expressed in CD4^+^ T lymphocytes cells which are inhibited in the heart after infarction of myocardium being the primarily target to A_2A_ agonist modulating a protective effect in heart (Yang et al., 2006). Furthermore, A_2A_ agonists reduce CD3^+^ T lymphocytes and neutrophils which could inhibit the cellular adhesion consequently no transmigration across endothelial cell occurs (Yang et al., 2005; Hasko and Pacher, 2008; Barletta et al., 2012). Macrophage is a defense cell that can also migrate to inflammatory location and its regulation might be through adenosine receptor activation. There are two phenotypes of macrophage: phenotype 1 (M1) which is characterized by expression of several inflammatory cytokines (TNF-α, IL-1β, IL-6, and IL-12) and chemokines ensuring an inflammatory profile and phenotype 2 (M2) which controls inflammation and improves tissue healing. Stimulation of A_2A_ receptors might promote the change in the macrophage phenotype 1 to type 2 (Hasko and Cronstein, 2013; Cronstein and Sitkovsky, 2017) promoting reduction of inflammation.

The A_2B_ receptors are preferentially coupled to stimulatory G protein (Fredholm et al., 2001), but also couple to Gq protein leading to the regulation of intracellular calcium levels (Feoktistov and Biaggioni, 1997; Linden et al., 1999). Activation of A_2B_ receptors might mediate mast cell function, increasing degranulation and stimulation of IL-8 secretion (Feoktistov and Biaggioni, 1995, 1996, 1997). In addition, the A_2B_ receptors activation increases IL-6 production by pulmonary fibroblasts, leading to the formation of myofibroblasts that deposit extracellular matrix (Hasko et al., 2008). In contrast, A_2B_ receptors might modulate beneficial anti-inflammatory effect through the inhibition of neutrophils activity (Barletta et al., 2012). Similarly to A_2A_ receptors, A_2B_ receptors when stimulated produce a change in the macrophage profile from phenotype 1 to phenotype 2.

The A_3_ receptors are widely expressed on immune cells and, upon binding their agonists, can activate phospholipase C and mediate inhibition of the PI3K/Akt and NF-kB signaling pathways to suppress production of TNF-α, IL-12, and IL-6 (Fishman et al., 2006, 2012; Varani et al., 2010b; Lee et al., 2011; Faas et al., 2017).

## RV Failure in PAH and Cardiac Profile of ARs

In an important review, Voelkel et al. (2006) described the most relevant structural and functional changes to occur in the RV chamber subsequent to long-term pressure overload due to elevated PVR. The initial response of the right heart is myocardial remodeling and hypertrophy to compensate for the elevated postload, accompanied by progressive contractile dysfunction. Finally, the chamber dilates to allow compensatory preload and to maintain stroke volume despite the reduced systolic function. Patients may develop clinical evidence of RV failure, including elevated filling pressure, diastolic dysfunction, and reduced cardiac output. The LV develops diastolic dysfunction due to the increased size and pressure overload of the RV, such that PAH results in global heart failure (Voelkel et al., 2006). Maladaptive RV remodeling and subsequent hypertrophy might be accelerated by neurohormonal signaling, oxidative stress of cardiac cells, metabolic changes, and myocardial inflammation, which affect the cardiomyocytes, cardiac fibroblasts, and coronary vascular cells (Ryan and Archer, 2014). Thus, we propose that a cardioprotective target should be beneficial by acting throughout all of the cardiac cells.

In the heart, adenosine might modulate the growth and death of cardiomyocytes, cardiac fibroblasts, ECs and SMCs, as well as affect the extracellular matrix (Headrick et al., 2013). One important study evaluated the impact of chronic heart failure on the adenosine system and the effects of its stimulation on disease development in humans.

Patients with moderate heart failure who were treated with dipyridamole, an adenosine uptake inhibitor, exhibited reduced AR expression, increased adenosine levels, and symptom improvement (Asakura et al., 2007). Hence, disturbances of the adenosine system might contribute to development of chronic heart failure.

Although preclinical and clinical studies have shown benefits of activating the A_1_ receptor in the context of LV dysfunction and failure (Ely and Berne, 1992; Headrick et al., 2003; Liao et al., 2003; Peart and Headrick, 2007; Chuo et al., 2016; Dinh et al., 2017), there is a lack of data regarding the effects of this receptor subtype in experimental models of PAH-induced RV impairment and global heart failure. We may discuss that the current unclear specific roles of this adenosine receptor subtype in the context of PAH should be the cause of researchers to consider it of low relevance.

Adenosine plays roles in the inflammatory process of LV heart failure. In cardiomyocytes from patients with LV heart failure, treatment with adenosine or the selective A_2_ receptor agonist DPMA decreased TNF-α expression levels by 40% or 87%, respectively (Wagner et al., 1998). Activation of the A_2A_ receptor mediated the inflammatory process via activation of PKA, which, in turn, inhibited synthesis of proinflammatory molecules, such as TNF-α and IL-1β (Varani et al., 2010a; Impellizzeri et al., 2011).

The A_2A_ receptor is expressed in mast cells (Marquardt, 1994), neutrophils (Fredholm et al., 1996), and CD4^+^ T cells (Koshiba et al., 1999). Xu et al. (1996) showed that adult rat ventricular myocytes express adenosine A_2A_ receptor messenger RNA and through an immunoblotting technique, it was demonstrated that they express A_2A_ receptor protein (Kilpatrick et al., 2002). Although numerous *in vivo* studies of the A_2A_ receptor implicate its anti-inflammatory effects, some studies have suggested that its cardioprotective activities could be due, at least in part, to direct myocardial effects (Woodiwiss et al., 1999; Monahan et al., 2000; Chandrasekera et al., 2010; McIntosh and Lasley, 2012). Constitutive overexpression of the A_2A_ receptor in young mice with LV dysfunction was associated with increases in cardiac contractility, heart rate, and LV mass (Chan et al., 2008).

Cardiac overexpression of the A_2A_ receptor showed protective effects by attenuating fibrosis and improving cardiac function in a mouse model of heart failure (Hamad et al., 2012). Another work showed that this receptor is expressed in cardiac fibroblasts, where it has an antifibrotic role (Sassi et al., 2014).

As addressed earlier in this review, our laboratory has synthesized two new molecules, LASSBio-1359 and LASSBio-1386, from the lead compound LASSBio-294. Using *in silico* approaches, we identified a putative docking pose of this compound class at the A_2A_ receptor. *In vitro* assays of all three compounds indicate A_2A_ receptor agonist activity, although second messenger assays and full binding curves were not performed. The compounds exhibited vascular benefits and cardioprotective activities when administered chronically in rats with MI-induced LV heart failure (Costa et al., 2010; da Silva et al., 2014, 2017) or PAH-induced RV heart failure (Alencar et al., 2013, 2014). LASSBio-294 was chronically administered to normotensive and spontaneous hypertensive rats 4 weeks after MI. In addition to showing inotropic and lusitropic activities, this drug decreased cardiac remodeling, reduced cell infiltration, and improved Ca^2+^ influx into the sarcoplasmic reticulum in two different studies (Costa et al., 2010; da Silva et al., 2017). In a third work, chronic administration of LASSBio-294 to rats 4 weeks after MI reduced exercise intolerance by recovering Ca^2+^ homeostasis in the skeletal muscle. LASSBio-294 prevented cellular infiltration into the skeletal muscle, similarly to what was described for cardiac muscle. We have proven that the beneficial effects of LASSBio-294 occur through its activation of the A_2A_ receptor (da Silva et al., 2017) in the cardiac and skeletal muscles (da Silva et al., 2014) and the subsequent increase of cAMP levels.

Given its salutary effects in animal models of left heart disease, we hypothesized that the A_2A_ receptor should show cardioprotective potential in both LV and RV tissues. We chronically administered our new adenosine A_2A_ agonists LASSBio-294 (data not published) LASSBio-1359 and LASSBio-1386 to rats with monocrotaline-induced RV failure (Alencar et al., 2013, 2014). Our new derivatives showed beneficial effects on the regulation of right heart physiology, as depicted by echocardiographic evaluations after long-term A_2A_ receptor activation (2 weeks of oral treatment with both substances) in monocrotaline-induced RV failure. Treatment of RV failure rats with LASSBio-1386 significantly improved their exercise capacity compared to control animals. Finally, we demonstrated, for the first time, that rats with RV failure had reduced A_2A_ receptor levels in RV tissue, which were correlated with reductions of SERCA2 content and Ca^2+^-ATPase activity. Activation of SERCA2 is one of the most important processes to regulate cardiomyocyte relaxation (Katz, 2006). SERCA induces Ca^2+^ uptake from the cytosol to the sarcoplasmic reticulum lumen, activating cell relaxation and promoting restock of Ca^2+^ for the next cardiac contraction. Elevated SERCA2 activity and density lead to an increased potential for the next cardiac cycle (Periasamy and Huke, 2001). These findings suggest the beneficial effects of A_2A_ receptor-mediated signaling on global cardiac function. Using echocardiography, we confirmed the impairment of systolic function in PAH rats (Alencar et al., 2014). These data are consistent with previous reports showing an impaired pattern of this receptor in different experimental models of LV disease.

Additionally, we may discuss that the reduced expression of A_2A_ receptor in the cardiopulmonary tissues in rats with PAH-induced RV failure may be a consequence of the chronic state of the disease. Possibly, in earlier stage of PAH, the A_2A_ receptor expression could be unchanged or increased as a physiological compensatory mechanism. However, further studies are necessary to determine this receptor subtype levels during all stages of the disease in preclinical models. Despite the reduced expression of the A_2A_ receptor in the cardiopulmonary system on late stage of PAH, lower levels of the receptor were still detected and LASSBio-1359 and LASSBio-1386 could bind and activate these receptors, ameliorating PAH.

No study has investigated whether the A_2B_ receptor plays a role in RV function or in the pathophysiology of PAH-induced RV failure. However, this receptor is highly expressed in cardiac fibroblasts. Despite displaying antifibrotic potential in several *in vitro* studies, most *in vivo* animal models have described this receptor as being an inducer of cardiac remodeling and fibrosis. In their review, Vecchio et al. (2017) proposed that some proinflammatory mechanism may underlie this profibrotic activity of the A_2B_ receptor. A_2B_ receptor promoted a moderate increase in cardiac contractility in *ex vivo* mouse hearts (Chandrasekera et al., 2010). A later study showed that A_2B_ receptor prevented mitochondrial oxidative stress by decreasing superoxide generation, but its effects were simultaneously potentiated by activation of the A_2A_ receptor. Xu et al. (2017) concluded that the A_2A_ and A_2B_ receptors act together in the regulation of heart metabolism.

Given its high expression in mast cells, neutrophils, eosinophils, and other inflammatory cells, A_3_ receptor has been speculated as a potential target for the treatment of ischemic conditions, glaucoma, asthma, arthritis, cancer, and other inflammation-related disorders (Gessi et al., 2008). Low-level expression of A_3_ receptor in the heart provided effective protection against ischemic injury without detectable adverse effects, whereas overexpression led to development of dilated cardiomyopathy. Expression of this receptor was below the limits of detection of radioligand binding or northern blot (Black et al., 2002). Combined with the lack of works addressing the potential of A_3_ receptor in PAH and RV failure, we do not have enough arguments to assume that the synthesis of new ligands for this adenosine target could represent a suitable strategy to treat the disease in the future.

## Conclusion

Over the past 20 years, scientists in medicinal chemistry have generated agonists and antagonists with high affinity and high selectivity for human variants of each of the four ARs. Moreover, agonist and antagonist ligands containing positron-emitting radioisotopes have been developed to monitor the *in vivo* occupancy of ARs in humans (Muller and Jacobson, 2011; Chen et al., 2013). As such, the lack of selective ligands of ARs is not a limiting factor for research and drug development, as has been the case for some other G-protein coupled receptors. Furthermore, researchers continue their efforts to develop novel adenosine ligands with refined structure-activity relationships, improved *in vivo* biodistribution, and tissue selectivity, which are crucial to druggability (Muller and Jacobson, 2011; Chen et al., 2013). A bigger problem in this process has been the broad distribution of the ARs. A possible approach for achieving tissue selectivity could be the use of partial agonists that would predominantly act where there are a high number of so-called “spare” receptors (Chen et al., 2013), as we also commented earlier in this review.

Advances in our understanding of the pathogenetic role of adenosine in PAH may soon be translated into effective treatment options. There is a complex interplay among the different distribution patterns and/or affinities of the four AR subtypes in specific cell types at different stages of disease. As such, combinations of selective antagonist/agonists for the different AR subtypes will likely be required to obtain reasonable clinical efficacy. Alternatively, controlling the factors involved in driving adenosine concentrations in tissue may be important.

Regarding the roles of A_1_, A_2B_, and A_3_ adenosine receptor subtypes, with this review we are assuming the need of closely studying each one in specific pre-clinical models of PAH for further discussions on their use as targets to treat this deleterious cardiopulmonary disease in the clinical field.

Nevertheless, data discussed in this review indicate a role for the A_2A_ receptor in mediating beneficial effects, such as pulmonary vascular relaxation, reduction of pulmonary vessel and RV hypertrophy, amelioration of RV dysfunction, and exercise capacity, in rats with PAH. We have briefly described the importance of the adenosine system, specifically the A_2A_ receptor, as a new target for the treatment of PAH. Several factors remain to be explored. For example, global and local A_2A_ receptor function should be investigated in human PAH. The site of action of these drugs should be carefully determined through their administration to animals with cell-specific receptor deletions. Adenosine signaling and the effects of each drug over the specific disease course (e.g., acute and chronic stages of PAH and RV failure) should be carefully monitored in clinical studies. Finally, the possibility of combining direct A_2A_ receptor actions with drugs targeting other pathways and/or targets should be examined. Such explorations could uncover new therapeutic strategies, which are greatly needed for patients with PAH.

## Author Contributions

All authors listed have made a substantial, direct and intellectual contribution to the work, and approved it for publication.

## Conflict of Interest Statement

The authors declare that the research was conducted in the absence of any commercial or financial relationships that could be construed as a potential conflict of interest.
